# Detection of Soluble Solids Content in Different Cultivated Fresh Jujubes Based on Variable Optimization and Model Update

**DOI:** 10.3390/foods11162522

**Published:** 2022-08-20

**Authors:** Haixia Sun, Shujuan Zhang, Rui Ren, Jianxin Xue, Huamin Zhao

**Affiliations:** College of Agricultural Engineering, Shanxi Agricultural University, Jinzhong 030801, China

**Keywords:** cultivation, visible/near infrared spectrum, fresh jujube, model update, variable fusion

## Abstract

To solve the failure problem of the visible/near infrared (VIS/NIR) spectroscopy model, soluble solids content (SSC) detection for fresh jujubes cultivated in different modes was carried out based on the method of variable optimization and model update. Iteratively retained informative variables (IRIV) and successive projections algorithm (SPA) algorithms were used to extract characteristic wavelengths, and least square support vector machine (LS-SVM) was used to establish detection models. Compared with IRIV, IRIV-SPA achieved better performance. Combined with the offset properties of the wavelength, repeated wavelengths were removed, and wavelength recombination was carried out to create a new combination of variables. Using these fused wavelengths, the model was recalibrated based on the Euclidean distance between samples. The LS-SVM detection model of SSC was established using the update method of wavelength fusion-Euclidean distance. Good prediction results were achieved using the proposed model. The determination coefficient (R^2^), root mean square error (RMSE), and residual predictive deviation (RPD) of the test set on SSC of fresh jujubes cultivated in the open field were 0.82, 1.49%, and 2.18, respectively. The R^2^, RMSE, and RPD of the test set on SSC of fresh jujubes cultivated in the rain shelter were 0.81, 1.44%, and 2.17, respectively. This study realized the SSC detection of fresh jujubes with different cultivation and provided a method for the establishment of a robust VIS/NIR detection model for fruit quality, effectively addressing the industry need for identifying jujubes grown in the open field.

## 1. Introduction

Containing various types of ingredients (such as sugars, vitamin C, and minerals), “Huping” jujube has high nutritional and medicinal values. The content of soluble solids (SSC) is an important evaluation index for the internal quality of fruit and vegetables, which is closely related to improving the added value of products and meeting consumer needs [1,2]. In traditional detection of SSC, destructive or invasive methods (e.g., refractometers) were used, which damaged the integrity of the sample and were cumbersome, time-consuming, and laborious to perform. This destructive approach is unfavorable for large-scale collection, implementation assessment, and industrial applications. Therefore, it is important to achieve rapid, non-destructive detection of SSCs to support the quality assessment and grading of agricultural products.

Visible/near infrared spectroscopy (VIS/NIR) [3,4] uses absorption characteristics of the frequency doubling and combined frequency absorption of hydrogen-containing groups (such as C-H, N-H, and O-H) to obtain characteristic information of samples, which realizes the detection of key chemical components and physical properties. Compared with traditional detection methods, VIS/NIR technology requires little or no sample preparation and has the characteristics of rapidity, non-destructiveness, real-time application, and low cost. It has been widely applied in the quality detection of agricultural products, such as fruits [5], vegetables [6], cereals [7], and pulses [8]. VIS/NIR spectroscopy is multivariate and contains multiple overlapping peaks related to compounds such as water, sugars, and proteins. The prediction accuracy of the VIS/NIR model was affected by some conditions such as samples (for example, maturity, variety, season, year, and batch) [9,10,11], instruments [12,13], and environment (for example, temperature) [14,15]. Developed models based on VIS/NIR spectral data were generally applicable to the quality detection of samples in a single condition. There is some variability in measured values under the new conditions. Modeling based on the data from the first condition does not involve this variability, and these models are usually not robust for the actual variability. For samples of different conditions, models built with a single condition perform poorly, and the bias and error are generally high.

The damage [16], pest [17], crack [18], SSC [19], and hardness [20] have been carried out in the quality detection of fresh jujubes using VIS/NIR spectroscopy. Those quality detections were implemented in the open-field cultivation mode, and the predicted samples had similar characteristics to those modeling samples. In addition to the open-field cultivation mode, there is also a rain-shelter cultivation mode that adopts the method of building a rainproof shed in the actual “Huping” jujube cultivation [21]. The rain-shelter method can avoid direct contact between rainwater and jujube fruit; reduce the impact of cracking, diseases, and insect pests on jujubes; and have good ventilation performance. Due to the differences in temperature, humidity, and solar radiation between rain-shelter and open-field cultivation, various internal component contents of different cultivated fruit are different, such as pear [22,23], cherry [24], and grape [25]. Inside the samples, the chemical composition is related to its optical absorption properties, and the physical structure is related to its scattering properties. Changes in the texture and internal component contents lead to different optical responses, which would affect the performance of the model built in spectral detection [26,27]. In the above studies of quality detection, spectral detection models were mainly developed for samples cultivated in open fields. However, the analysis of this model prediction performance for fresh jujubes from different cultivation modes is rarely reported.

Several studies have been reported to address the poor performance of models constructed from a single condition. Mishra et al. [28] updated the NIR detection models of the moisture content and SSC for pears, which significantly improved the prediction results for samples of different batches. Sun et al. [29] pointed out that temperature had an influence on the spectral detection model of mango dry matter content and established a robust prediction model using the method of temperature correction. For mango dry matter content based on multi-season, multi-variety, and multi-growing regions, Anderson et al. [30] established a robust prediction model. In order to reduce the influence of instruments, seasons, and temperature changes on the fruit NIR detection model in the study of Mishra et al. [31], calibration models that preserved performance under new conditions were established. The above studies showed that reference measurements from new conditions were generally required in order to compensate for external influences. Model updating was an important method of resolving poor performance when the VIS/NIR model was applied to new conditions. New samples were required in the model update, and the method for determining the number of new samples needed to be investigated. In addition, a reasonable selection of variables could reduce the influence of interfering information and improve model performance due to the high dimensionality and overlapping peaks of VIS/NIR data. There are few studies incorporate wavelength offset properties into the selection of variables.

Therefore, the objective of this study was to develop a robust model for the SSC detection of fresh jujubes from different cultivation modes based on VIS/NIR spectroscopy. To achieve this aim in this study, the IRIV-SPA was used to select characteristic wavelengths, and a new combination of variables was established in combination with wavelength position offset properties. A model update using wavelength fusion-Euclidean distance was proposed to re-calibrate the SSC model. The proposed method achieves the SSC prediction of fresh jujubes from different cultivation modes and improves the generalizability and stability of the model.

## 2. Materials and Methods

### 2.1. Sample Collection

The two methods are applied to the planting of “Huping” jujube. The rain shelter was built in the immature period of jujube fruit. The top is covered with plastic film, and the surrounding is ventilated. The full-maturity “Huping” jujubes cultivated in the open field and the rain shelter were collected from an orchard in Taigu, China, respectively. On the same day of collection, samples were transported to the laboratory, cleaned, and placed for four hours to return to room temperature. In this study, a total of 300 intact samples (150 rain-shelter samples and 150 open-field samples) were selected. For each cultivation, the KS algorithm [32] was used to divide the data set into a calibration set (114 samples) and a prediction set (36 samples) with a ratio of 3:1.

### 2.2. Spectrum Acquisition and SSC Determination

**Figure 1:**
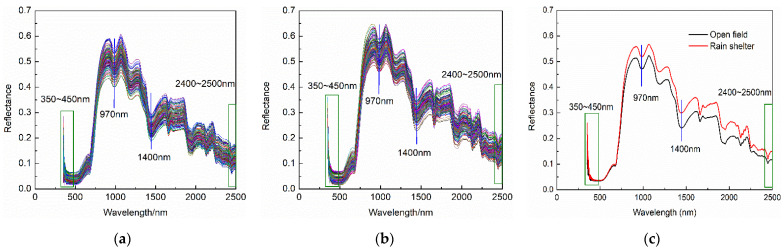
Mean spectrum of fresh jujubes. (**a**) Spectral curves of open-field cultivation, (**b**) spectral curves of rain-shelter cultivation, and (**c**) average spectrum.

Spectrum data were collected using an ASD Fieldspec3 spectrometer (analytical spectral device, Longmont, CO, USA) with spectral resolutions of 3 nm@350~1000 nm and 10 nm@1000~2500 nm and a spectral range of 350~2500 nm. The spectral curves of fresh jujubes cultivated in the open field and the rain shelter are shown in Figure 1. Although there were some differences in reflectance values of fresh jujubes between the two cultivation modes, the curve-change trends of the two cultivation methods were relatively similar. There were obvious absorption peaks related to the stretching vibration of O-H bond at the vicinity of 970 nm and 1400 nm. The signal-to-noise ratio was low, and the noise was loud at 350~450 nm and 2400~2500 nm. Therefore, the spectral information of 450~2400 nm was selected for subsequent analysis.

The SSC of each sample was measured using a hand-held refractometer. The statistical values are shown in Table 1. The SSCs of fresh jujubes cultivated in the open field and rain shelter were 21.2–35.5% and 21.8–37.4%, respectively.

### 2.3. Data Analysis

In this study, the baseline and Savitzky Golay (SG) were used for the preprocessing, respectively. Spectral preprocessing was performed using Unscrambler X10.1 software. The IRIV and SPA were used to extract characteristic wavelengths, and the LS-SVM was adopted to build the model. LS-SVM [33,34] follows the principle of structural risk minimization and transforms the convex quadratic programming problem of traditional support vector machines into the problem of solving a system of linear equations, which reduces the computational complexity. Variable extraction of IRIV and SPA and modeling of LS-SVM were carried out using MATLAB R2020a. The determination coefficient (Rc^2^) and the root mean square error (RMSEC) of the calibration set, the determination coefficient (Rp^2^) and the root mean square error (RMSEP) of the prediction set, and the residual predictive deviation (RPD) were used to evaluate the model performance.

### 2.4. Data Processing Method

#### 2.4.1. Wavelength Extraction Method

The SPA [35,36] eliminates the influence of collinearity among various variables, reduces the overlap of effective information, and accelerates the modeling speed.

The IRIV method [37] utilizes random combinations of variables and interactions between variables to select variables based on binary matrix rearrangement filters. Based on the model cluster analysis method, the difference of mean values (DMEAN) and the P value were calculated to determine the class of each variable. The classification rules of variables are shown in Table 2.

#### 2.4.2. Model Update Method

To improve the applicability of the model, a sample addition algorithm was proposed to update the model. Combined with wavelength position offset properties, wavelength fusion was performed. The KS algorithm calculates the Euclidean distance and adds the two samples with the largest distance to the calibration set. Then, the distance minimum (Dm) value between each remaining sample and the selected calibration set sample is calculated. The sample with the largest Dm is added to the calibration set until the calibration set reaches the specified number of samples. Therefore, samples with large spectral differences between different cultivation modes can be extracted for use as a calibration set using the KS algorithm. In this study, based on the Euclidean distance between samples, the KS algorithm was used to select new samples in turn, and the RMSECV of the established PLSR model was calculated.

## 3. Results and Discussion

### 3.1. Establishment of SSC Detection Model for Fresh Jujubes in Open-Field Cultivation

#### 3.1.1. SSC Detection Models Using Full Wavelengths

Open-field cultivation is the main mode in the planting of jujube. The spectral information of the open-field cultivation samples was pre-processed using baseline and SG. Based on the spectrum with no-pretreatment and pretreatment, LS-SVM models of the SSC were established, and fresh jujubes from two cultivation modes were predicted. The prediction results are shown in Table 3.

In Table 3, when built models with the samples cultivated in the open field were used to predict the samples of the same cultivation methods, results of no-pretreatment and SG pretreatment were similar (Rp^2^ = 0.80, RMSEP = 1.14%, RPD = 2.25) and better than the results of baseline. The prediction ability of the three methods for rain-shelter samples was not ideal (Rp^2^ = 0.47~0.59, RMSEP = 2.54~2.77%, RPD = 1.03~1.12), which indicated that the sharing ability of the model needs to be improved. On the whole, compared with the results of baseline and SG, the prediction ability of the constructed model with no-pretreatment was better. Therefore, spectral data without preprocessing were used for analysis in the following study.

#### 3.1.2. Establishment of an SSC Model Using the IRIV-SPA

**Figure 2:**
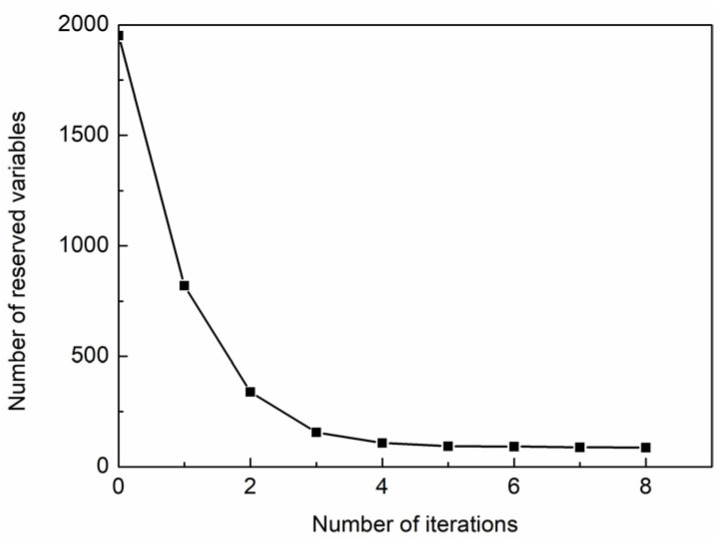
Number of retained variables.

**Figure 3:**
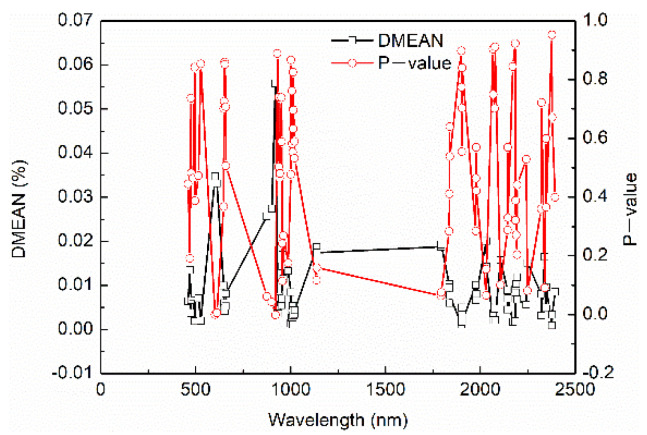
DMEAN and P–values of the nonparametric Mann–Whitney U test on variable.

Based on the spectral data of fresh jujubes in the open field, the IRIV algorithm with an inverse elimination was used to select variables. The number of cross-validation, maximum principal component, and iteration were set as 10, 20, and 8, respectively. In this IRIV iterative process, the change curve of the retained variable number is shown in Figure 2. As the iteration number increased, the number of retained variables decreased, and the downward trend gradually flattened. Overall, 87 wavelength variables were retained at the 8th iteration. The DMEAN and P –values of retained variables are shown in Figure 3.

**Figure 4:**
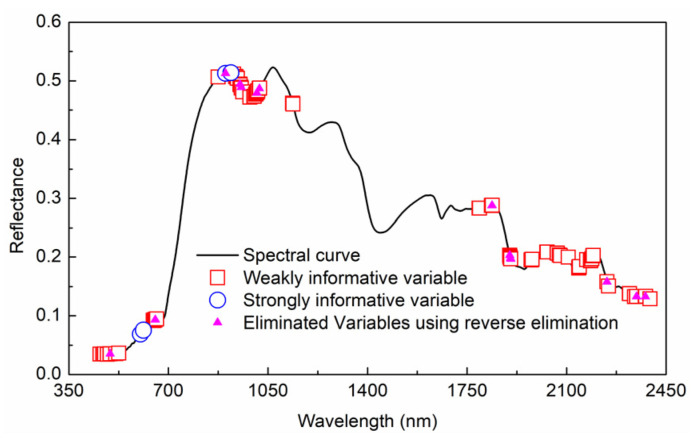
Selection of characteristic wavelength using IRIV based on open-field cultivation.

Combining Figure 3 and the rules in Table 2, the variable type was divided. The selected variables and types are shown in Figure 4. In total, 4 strong informative variables and 83 weak informative variables were selected from 1951 variables, respectively. An inverse elimination was performed, and 71 characteristic wavelengths were preserved. 

The number of selected characteristic wavelengths using the IRIV algorithm was high. For further data dimensionality reduction, SPA was used to perform an extraction of characteristic wavelengths for the second time based on 71 extracted characteristic wavelengths using IRIV. When the RMSE was 1.0257%, 10 characteristic wavelengths were extracted. According to the degree of importance, the extracted wavelengths using IRIV-SPA were 957, 1008, 2339, 920, 2248, 2394, 1137, 1976, 647, and 602 nm in turn.

Based on extracted characteristic wavelengths using IRIV and IRIV-SPA, LS-SVM was used to establish SSC detection models. The SSC of fresh jujubes from two cultivation modes was predicted. The SSC-predicted results are shown in Table 4.

Compared with the built model using full wavelengths (in Table 3), the IRIV-LS-SVM model improved the prediction ability. For the SSC of open-field samples, the Rp^2^ (from 0.80 to 0.85) and the RPD (from 2.25 to 2.52) were increased, and the RMSEP decreased from 1.14% to 1.02%. For the SSC of rain-shelter samples, the Rp^2^ (from 0.59 to 0.71) and the RPD (from 1.12 to 1.14) were increased, and the RMSEP decreased from 2.54% to 2.50%. For the prediction results of open-field samples, the IRIV-SPA-LS-SVM model and the full-wavelength LS-SVM model were basically the same. For the prediction results of samples cultivated in the rain shelter, the IRIV-SPA-LS-SVM model was slightly worse than the full-wavelength LS-SVM model. Based on the IRIV-LS-SVM and IRIV-SPA-LS-SVM models of fresh jujubes cultivated in the open field, the SSC of samples in the same cultivation was well-predicted, but the SSC of samples in the rain-shelter cultivation was poorly predicted. Therefore, the cultivation mode has a certain influence on the SSC detection model, and the model needs to be further optimized to improve the predictive ability. Compared with IRIV, the number of extracted characteristic wavelengths using IRIV-SPA was significantly reduced (from 71 to 10) on the premise of ensuring the model performance. The IRIV-SPA algorithm achieved a better comprehensive ability and was used to select the characteristic wavelength in the following research.

### 3.2. Update of SSC Detection Model

#### 3.2.1. Variable Optimization

**Figure 5:**
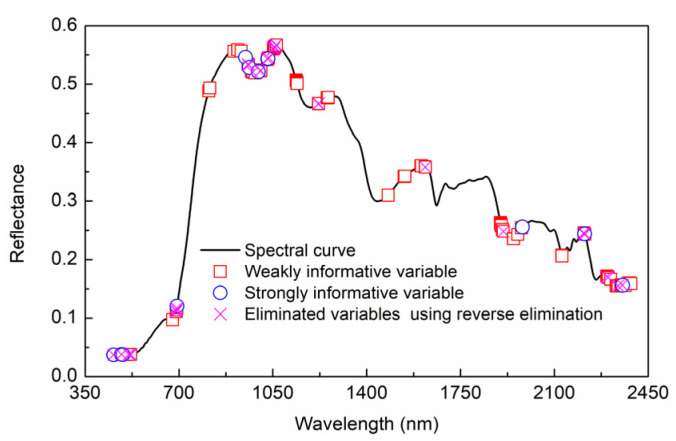
Selection of characteristic wavelength using IRIV based on rain-shelter cultivation.

Based on the spectral information of samples cultivated in the rain shelter, the selection of SSC characteristic wavelengths using IRIV is shown in Figure 5. Thirteen strongly informative variables and ninety-three weakly informative variables were selected. After reverse elimination of variables, the final number of optimal characteristic wavelengths was 73. 

Based on the extracted characteristic wavelengths using IRIV, SPA was used to extract the characteristic wavelengths for the second time. Ten characteristic wavelengths were extracted when the RMSE was 1.0257%. According to the importance, the extracted characteristic wavelengths using IRIV-SPA were 1257, 962, 905, 1137, 2337, 2300, 1541, 2378, 2386, 1947, 1907, 1480, 1058, 2128, 811, and 693 nm in turn. 

**Figure 6:**
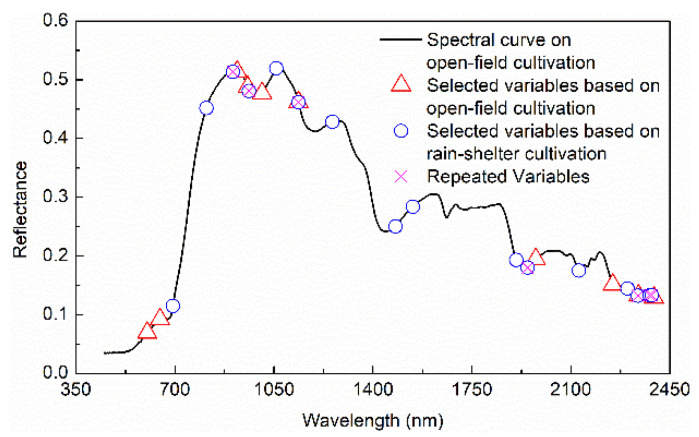
Fusion of characteristic wavelength.

For fresh jujubes from two cultivation modes, the selected characteristic wavelengths using IRIV-SPA are shown in Figure 6. There was a certain difference between the extracted characteristic wavelengths using the two cultivation modes. For the characteristic wavelengths extracted from a single cultivation mode, it was difficult to cover up the characteristic information of another cultivation mode.

Therefore, a new variable combination that integrated the extracted characteristic wavelengths of open-field and rain-shelter cultivation was proposed. In Figure 6, there were also the same and similar wavelengths between the characteristic wavelengths of those two cultivation modes. The selected variables of the two cultivation modes were added, redundant repeat variables were removed from the added characteristic variables, and the remaining variables were used as the fused characteristic wavelengths. Due to differences in the physicochemical properties of the sample, the external environment, and other factors, there would be a certain positional shift between wavelengths [38,39,40]. In this study, the wavelength corresponding to the position shift in the range of (−30 nm, 30 nm) was used as a repeated variable. Only one variable remained among the repeated variables, and redundant variables were removed. The extracted SSC characteristic wavelengths after fusion (in Figure 6) were 602, 647, 693, 811, 920, 957, 1008, 1058, 1137, 1257, 1480, 1541, 1907, 1976, 2128, 2248, 2300, 2339, and 2394 nm.

#### 3.2.2. Model Update

**Figure 7:**
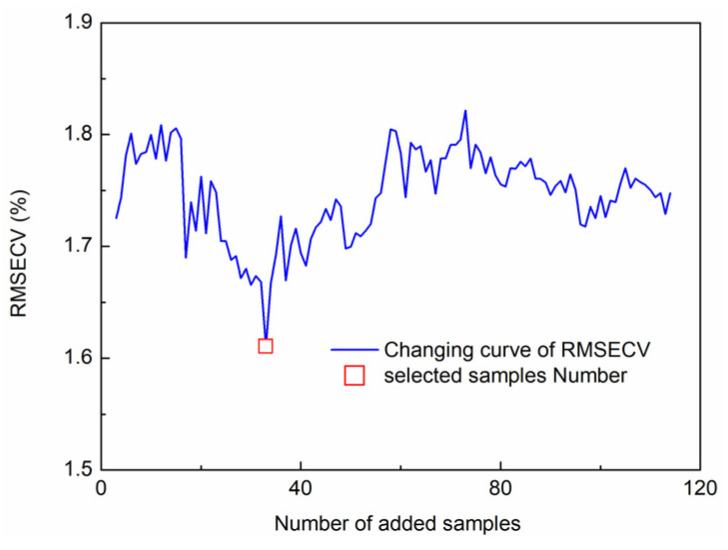
RMSECV distribution in different numbers of samples.

Because of the difference between the spectral curves of the two modes, the Euclidean distance between full wavelength spectrum of samples from the rain shelter. The KS algorithm was used to sequentially select samples from the calibration set of fresh jujubes cultivated in the rain shelter, sequentially. The new selected samples were added to the calibration set of fresh jujubes cultivated in the open field to form an updated calibration set, sequentially. PLSR was adopted to establish SSC detection models based on the updated calibration set, and the minimum value of RMSECV was used as the rule for selecting samples. The changing curve of RMSECV for SSC is shown in Figure 7. The minimum value of RMSECV was 1.33%. Correspondingly, 33 samples were selected from the calibration set of fresh jujubes cultivated in the rain shelter.

Thirty-three selected samples from rain shelter cultivation were combined with the calibration set from open-field cultivation (114 samples) to form an updated calibration set (147 samples). Based on the fused characteristic wavelengths, the original calibration set and the updated calibration set were used to establish LS-SVM detection models, respectively. The predicted results are shown in Table 5.

For the prediction ability (in Table 5) of fresh jujubes from two cultivation modes, the established model based on wavelength fusion was better than the model established before updating. This indicated that the updated characteristic wavelengths after wavelength fusion did not interfere with the performance of the established model on the open-field cultivation. For the SSC prediction results of fresh jujubes cultivated in the open field, the two update methods were good and similar. For the SSC prediction results of fresh jujubes cultivated in the rain shelter, the updated model based on wavelength fusion-Euclidean distance (Rp^2^ = 0.81, RMSEP = 1.35%, RPD = 2.10) was significantly better than the updated model based on wavelength fusion (Rp^2^ = 0.69, RMSEP = 2.96%, RPD = 0.96). Compared with the prediction performance before the model update, the ability of the updated LS-SVM model with the wavelength fusion-Euclidean distance was significantly improved. For fresh jujubes cultivated in open field, the Rp^2^ (0.79) was the same, the RPD increased from 2.14 to 2.20, and the RMSEP decreased from 1.20% to 1.17%. For fresh jujubes cultivated in the rain shelter, the Rp^2^ (from 0.65 to 0.81) and the RPD (from 0.85 to 2.10) were significantly increased, and the RMSEP (from 3.33% to 1.35%) decreased significantly. Therefore, the LS-SVM model based on the updated method of wavelength fusion-Euclidean distance achieved the best SSC prediction for fresh jujubes in both cultivation modes. 

**Figure 8:**
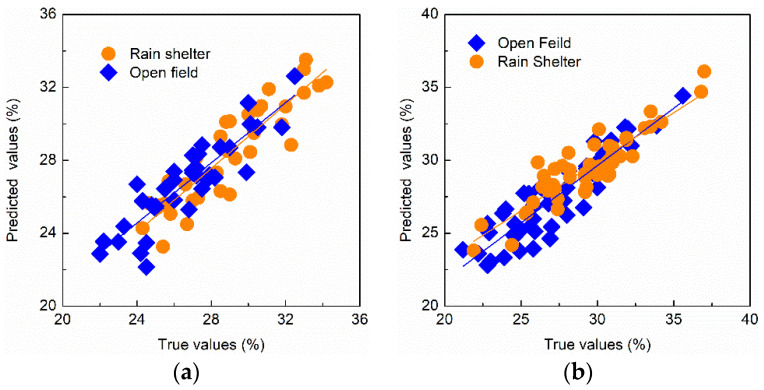
Detection results of SSC after model update using wavelength fusion-Euclidean distance. (**a**) Prediction set results; (**b**) test set results.

To validate the performance of the model based on the updated method of fusion wavelength-Euclidean distance, 50 samples from open-field cultivation and 50 samples from rain-shelter cultivation were collected for testing. To better show the detection results, the SSC-predicted and true values of samples from two cultivation modes are shown in Figure 8.

It was shown that the LS-SVM model using the update method of wavelength fusion-Euclidean distance achieved good prediction and test results for the SSC of fresh jujubes from both cultivation modes in Figure 8. The R^2^, RMSE, and RPD of the test set were 0.82, 1.49%, and 2.18 for the SSC of “Huping” jujubes from open-field cultivation, respectively. The R^2^, RMSE, and RPD of the test set were 0.81, 1.44%, and 2.17 for the SSC of “Huping” jujubes from rain-shelter cultivation, respectively.

In the field of fruit quality detection, a common problem was the failure of VIS/NIR spectral models. The established model has good prediction results under a single condition, but the model fails under new conditions with some variability. In the actual production of “Huping” jujube, there are two cultivation modes (open-field cultivation and rain-shelter cultivation). The established SSC detection model based on samples cultivated in open-field cultivation failed to predict samples cultivated in rain-shelter cultivation. In the SSC detection, there was some difference between the extracted characteristic wavelengths from the open-field samples and those from the rain-shelter samples. The dimensionality of the visible/NIR spectra was high and a direct replication of the VIS/NIR spectrum resulted in redundant information, which would affect model performance. In this study, the IRIV-SPA was used to preferentially select feature wavelengths that removed the effects of interfering information and uninformative variables. It was ensured that valid information was extracted, while the dimensionality was reduced. At the same time, variable recombination combined with wavelength position shift theory was used for variable selection. The preferred fusion wavelengths covered the variable information under the new conditions, which increased the coverage of feature information and did not interfere with the modeling ability of the original variables because of the new variables. When new samples were introduced based on Euclidean distances, the variability under the new conditions was increased. These variables were involved in the modeling when the model was recalibrated, which improved the accuracy and robustness of the model. Therefore, the wavelength fusion-Euclidean distance update method achieved good SSC prediction results for fresh jujube from two cultivation modes synchronously. The proposed method is an effective model updating method, which provides methods for the establishment of a robust VIS/NIR detection model and ideas for the online detection of agricultural product quality based on VIS/NIR spectroscopy.

## 4. Conclusions

In this study, the SSC detection of fresh jujubes cultivated in different modes (open-field cultivation and rain-shelter cultivation) was carried out based on VIS/NIR spectroscopy using variable selection and model updating. Based on the full-wavelength and the extracted characteristic wavelengths using IRIV and IRIV-SPA, the established LS-SVM models all achieved good predictions for the SSC of fresh jujubes cultivated in the open field, but the prediction results for samples cultivated in the rain shelter were all unsatisfactory. Compared with the IRIV algorithm, the IRIV-SPA algorithm achieved better performance. The extracted characteristic wavelengths of the two cultivation modes using IRIV-SPA were fused together. Combining the wavelength shift characteristics of the VIS/NIR spectrum, the repeated wavelengths were eliminated to form a new variable combination. Variable selection using wavelength fusion improved the SSC prediction results, but the degree of improvement in the SSC prediction of samples from rain-shelter cultivation needed to be increased. The method of adding samples under new conditions was applied for the model update. The updated LS-SVM model using the wavelength fusion-Euclidean distance achieved the best prediction results for SSC of fresh jujubes cultivated in the open field (Rp^2^ = 0.79, RMSEP = 1.17%, RPD = 2.20) and the rain shelter (Rp^2^ = 0.81, RMSEP = 1.35%, RPD = 2.10). The test results showed that the R^2^, RMSE, and RPD for the SSC of “Huping” jujubes from open-field cultivation were 0.82, 1.49%, and 2.18, respectively. The R^2^, RMSE, and RPD for the SSC of “Huping” jujubes from rain-shelter cultivation were 0.81, 1.44%, and 2.17, respectively. The method proposed in this study realizes the SSC detection of different cultivated fresh jujubes, provides a method for the establishment of a robust VIS/NIR detection model, and provides a basis for the online detection of fruit quality. In the future, a production line for the quality detection of fresh jujubes will be developed and optimized based on VIS/NIR spectroscopy.

## Figures and Tables

**Table 1:** Statistics of soluble solids content (%).

Cultivation	Data Set	Maximum	Minimum	Mean	Standard Deviations
Open field	Total samples	35.5	21.2	26.59	2.78
	Calibration set	35.5	21.2	26.55	2.86
	Prediction set	32.5	22	26.69	2.57
Rain shelter	Total samples	37.4	21.8	28.81	3.10
	Calibration set	37.4	21.8	28.75	3.18
	Prediction set	34.2	24.3	28.97	2.84

**Table 2:** Variable classification rules of iteratively retains informative variables (IRIV) [37].

Variable Class	Classification Rules
Interfering variable	DMEANi > 0, Pi < 0.05
Uninformative variable	DMEANi > 0, Pi > 0.05
Strongly informative variable	DMEANi < 0, Pi < 0.05
Weakly informative variable	DMEANi < 0, Pi > 0.05

**Table 3:** Prediction results of SSC using different pre-processing methods.

Pretreatment	Prediction Set	Rc^2^	RMSEC (%)	Rp^2^	RMSEP (%)	RPD
No-pretreatment	Open field	0.84	1.15	0.80	1.14	2.25
	Rain shelter			0.59	2.54	1.12
Baseline	Open field	0.83	1.24	0.67	1.48	1.74
	Rain shelter			0.47	2.77	1.03
SG	Open field	0.84	1.15	0.80	1.14	2.25
	Rain shelter			0.58	2.54	1.12

**Table 4:** Prediction results of SSC using IRIV and IRIV-SPA.

Variable Selection Methods	Number of Wavelengths	Prediction Set	Rc^2^	RMSEC (%)	Rp^2^	RMSEP (%)	RPD
IRIV	71	Open field	0.93	0.76	0.85	1.02	2.52
		Rain shelter			0.71	2.50	1.14
IRIV-SPA	10	Open field	0.82	1.23	0.79	1.20	2.14
		Rain shelter			0.65	3.33	0.85

**Table 5:** Prediction results of SSC after model update.

Model Update	Prediction Set	Rc^2^	RMSEC (%)	Rp^2^	RMSEP (%)	RPD
No update	Open field	0.82	1.23	0.79	1.20	2.14
	Rain shelter			0.65	3.33	0.85
Wavelength fusion	Open field	0.85	1.10	0.80	1.17	2.20
	Rain shelter			0.69	2.96	0.96
Wavelength fusion-Euclidean distance	Open field	0.88	1.02	0.79	1.17	2.20
	Rain shelter			0.81	1.35	2.10

## Data Availability

Data are contained within the article.
